# Differential effects on TDP-43, piezo-2, tight-junction proteins in various brain regions following repetitive low-intensity blast overpressure

**DOI:** 10.3389/fneur.2023.1237647

**Published:** 2023-10-09

**Authors:** Lanier Heyburn, Shataakshi Dahal, Rania Abutarboush, Eileen Reed, Rodrigo Urioste, Andrew Batuure, Donna Wilder, Stephen T. Ahlers, Joseph B. Long, Venkatasivasai Sujith Sajja

**Affiliations:** ^1^Blast Induced Neurotrauma Branch, Walter Reed Army Institute of Research, Silver Spring, MD, United States; ^2^The Geneva Foundation, Tacoma, WA, United States; ^3^Naval Medical Research Center, Silver Spring, MD, United States

**Keywords:** relBOP, blast, TDP-43, piezo, tight junction proteins

## Abstract

**Introduction:**

Mild traumatic brain injury (mTBI) caused by repetitive low-intensity blast overpressure (relBOP) in military personnel exposed to breaching and heavy weapons is often unrecognized and is understudied. Exposure to relBOP poses the risk of developing abnormal behavioral and psychological changes such as altered cognitive function, anxiety, and depression, all of which can severely compromise the quality of the life of the affected individual. Due to the structural and anatomical heterogeneity of the brain, understanding the potentially varied effects of relBOP in different regions of the brain could lend insights into the risks from exposures.

**Methods:**

In this study, using a rodent model of relBOP and western blotting for protein expression we showed the differential expression of various neuropathological proteins like TDP-43, tight junction proteins (claudin-5, occludin, and glial fibrillary acidic protein (GFAP)) and a mechanosensitive protein (piezo-2) in different regions of the brain at different intensities and frequency of blast.

**Results:**

Our key results include (i) significant increase in claudin-5 after 1x blast of 6.5 psi in all three regions and no definitive pattern with higher number of blasts, (ii) significant increase in piezo-2 at 1x followed by significant decrease after multiple blasts in the cortex, (iii) significant increase in piezo-2 with increasing number of blasts in frontal cortex and mixed pattern of expression in hippocampus and (iv) mixed pattern of TDP-3 and GFAP expression in all the regions of brain.

**Discussion:**

These results suggest that there are not definitive patterns of changes in these marker proteins with increase in intensity and/or frequency of blast exposure in any particular region; the changes in expression of these proteins are different among the regions. We also found that the orientation of blast exposure (e.g. front vs. side exposure) affects the altered expression of these proteins.

## 1. Introduction

Traumatic brain injury (TBI) resulting from blast exposure is a major concern among military and civilian personnel. Between 2000 and 2021, approximately 450,000 U.S military service members (SMs) were diagnosed with some form of TBI, including the most recent cases of TBI in 109 US troops after an attack on the Ain al-Asad airbase in Iraq in 2020 (1) and 23 cases of TBI during two attacks in March 2023 in Syria (2). Around 80% of them were diagnosed to be mild TBI (mTBI) (3) and the symptoms manifested around the immediate to acute timeframe after the attack. Most of these TBIs were shown to be associated with the blast by a retrospective study (4). In addition, many SMs are exposed to repetitive low-intensity blast overpressure (relBOP) from breaching and heavy weapons systems on multiple occasions throughout their careers, the adverse effects of which have been largely overlooked and, until recently, unstudied. The National Defense Authorization Acts (NDAAs) FY18 Sec 734, FY19 Sec 253 and FY20 Sec 717 mandated the study of BOP in training and combat environments (3, 5). There is a growing body of pre-clinical and clinical evidence that exposure to relBOP in training environments can potentially cause negative consequences such as headaches, altered cognitive status, and performance deficits at an acute stage (6–9). These acute changes have a potential risk of developing into long-term neurodegenerative cascades, causing lasting behavioral changes, including ear ringing, sleep disruptions, and memory problems (10–12).

In previous studies, we have shown that molecular expression patterns for the same markers are markedly different under (i) a range of injury conditions, including the presence of multi-organ injury in some situations and (ii) sub-threshold exposures or the exposures that are present in training environment with no obvious signs of lung injury (for this study it is 8.5 psi up to 30 frontal exposures) in others. In this context, we previously showed that levels of TDP-43, a very tightly regulated protein in the brain that is clinically connected to several neurodegenerative disorders such as Alzheimer’s disease, amyotrophic lateral sclerosis, and frontotemporal dementia (13), were differentially affected with varied numbers and magnitudes of blast exposure (14). In addition, we have found changes in tight junction-and blood–brain barrier-associated proteins such claudin-5, occludin and glial fibrillary acidic protein (GFAP) (11). Similarly, we also found that the expression of Piezo 2, a cation channel receptor involved in mechanotransduction, increased after multiple pressure blasts (14) suggesting a sensitivity to mechanical stimuli in the brain. This mechanotransduction protein could be one of the first responders to pressure from BOP potentially contributing to cellular changes (e.g., TDP-43 levels) following relBOP. However, due to structural and biomechanical heterogeneity of the brain, the pathological changes associated with the mechanical insult produced by relBOP may not be uniformly observed throughout the brain, thus affecting different regions of the brain and associated neuroanatomical structures differently. The goal of this study is to define injury risk associated with cumulative effects of relBOP exposure on various regions of the brain. We have also explored the cumulative effects of relBOP in different orientations to exposure.

## 2. Materials and methods

### 2.1. Animals

All animal experiments were conducted in accordance with the Animal Welfare Act and other federal statutes and regulations relating to animals and experiments involving animals and adhered to principles stated in the Guide for the Care and Use of Laboratory Animals (NRC Publication 2011 edition) using an Institutional Animal Care and Use Committee approved protocol. Two male Sprague Dawley rats, 8–9 weeks old that weighed ~275 g (Charles River Laboratories, Wilmington, MA) were housed at 20–22°C (12 h light/dark cycle) with free access to food and water *ad libitum* throughout the experiment with change in cages 2 times a week as per housekeeping guidelines of Walter Reed Army Institute of Research’s Veterinary Services Programs (VSP).

### 2.2. Blast overpressure exposure

Rats were anesthetized with isoflurane and subjected to repeated survivable blast overpressures (relBOP) using an ABS located at the Walter Reed Army Institute of Research (WRAIR). The ABS consists of a 0.5 ft. long compression chamber that is separated from a 21 ft. long transition/expansion test (15). The anesthetized rat was secured in the test section in a longitudinal (head-on; on-axis) or transverse (side-on; off-axis) orientation to the direction of blast exposure. The compression chamber was pressurized with room air, causing membranes to rupture at a pressure that is dependent upon the thickness of the specific membrane sheet separating the two chambers, yielding a supersonic blast wave that impacts the experimental subject in the test section. To yield relBOP in rats in these experiments, acetate membranes (Grafix Plastics, OH) were used to yield peak positive static pressures of approximately 4, 6.5 or 8.5 psig. Animals (*n* = 6 per group) were exposed to a single daily blast of 4 (impulse: 6.05 psig*msec), 6.5 (8.62 psig*msec), or 8.5 psig (impulse: 11.46 psig.msec) for either one (1x), four (4x), 14 (14x), or 30 (30x) days from the front or 30 (30x) days from the side; repeated blast exposures were separated by 24 h. Animals were randomly assigned to different blast exposure groups or the sham group to reduce variabilities from unforeseen confounding factors and ensure that any observed differences can be attributed to the blast regimen. All sham animals were subjected to isoflurane anesthesia, loaded in the shock tube, and underwent recovery procedures as described above, but were not exposed to blast overpressure (BOP). At 24 h following final BOP exposure, animals were euthanized with isoflurane overdose and the brain was dissected into distinct regions, including cortex, frontal cortex, and hippocampus. We used Paxinos atlas to macro-dissect the regions of the brain. Brain was sliced approximately 2 mm apart using a brain mold, we have then isolated frontal cortex from the bregma 2.20 mm to – 0.20 mm, cortex encompasses from Bregma −2.56 mm to −4.2 mm. The dissected tissues were then flash frozen on dry-ice and stored at −80°C until further analysis. Personnel for the analysis of samples was blinded to experimental conditions.

### 2.3. Protein extraction

Tissue was homogenized at 10% w/v in T-PER Tissue Protein Extraction Reagent (cat# 78510, ThermoFisher, NY) with 1% protease/phosphatase inhibitor cocktail (Sigma-Aldrich). Homogenate was centrifuged at 5000 × *g* for 5 min at 4°C. The supernatant, containing the soluble protein fraction, was collected, and stored at −80°C until use for Western blot.

### 2.4. Western blot

Western blot samples were prepared by mixing sample homogenates (~25 μg) with buffer containing loading dye to a volume of 20 μL, which was loaded into a NuPAGE™ 4–12% 1.0 mm, 12-well Bis-Tris Protein Gel (cat# NP0322BOX, ThermoFisher). Gels were run at 180 V for 35 min. Separated protein products were transferred to a PVDF membrane using the iBlot PVDF Transfer Stack and iBlot2 Dry Blotting System (ThermoFisher). The membranes containing protein products were blocked for non-specific binding for 1 h at room temperature using 2 g milk in 50 mL TBST buffer. The membranes were then incubated overnight with primary antibody for target protein and beta-actin at 4°C. After overnight incubation, the membraned were washed several times followed by 1 h incubation of HRP-linked secondary antibody at room temperature. Membranes were washed again before developing. Membranes were incubated for 5 min at room temperature in Pierce ECL Western Blotting Substrate (ThermoFisher) and were imaged using a FluorChem HD2 imager (Protein Simple) set at auto-exposure. Bands were analyzed by densitometry analysis using ImageJ software (NIH). Briefly, the area under the curve of pixel density of each target protein bands and their respective beta-actin bands were measured. The area of target protein was normalized to the area of beta-actin to obtain the final values. Primary antibodies included rabbit polyclonal antibody against TDP-43 (1:2000, ProteinTech cat# 10782-2-AP), rabbit polyclonal antibody against FAM38B/Piezo2 (1:2000, ProSci cat# 26–438), rabbit polyclonal antibody against GFAP (1,2000, Abcam, cat# ab7260), mouse monoclonal antibody against occludin (1,2000, ThermoFisher cat# 33–1,500), mouse monoclonal antibody against claudin-5 (1,2000, ThermoFisher cat# 35–2,500), and mouse monoclonal antibody against β-actin (1,20,000 abcam A2228). Secondary antibodies included goat anti-rabbit IgG (1,2,500, cat# 65–6,120) and anti-mouse IgG (1,2,500, Thermo cat# 32430).

### 2.5. Statistical analysis

All data were normalized relative to sham levels. A total of 6 animals per group (*n* = 6) was used. Number of animals per group was determined based on the power analysis of our previous study (11, 14) One-way ANOVA test was performed, with Dunnett’s *post hoc* test, for each protein. *p* < 0.05 was considered statistically significant. Unless otherwise specified, all data are expressed as mean ± SEM. All the results are expressed as 1-time exposure as 1x, 4-time exposure as 4x, 14-time exposure as 14x and 30-time exposure as 30x.

## 3. Results

### 3.1. Occludin

**Figure 1:**
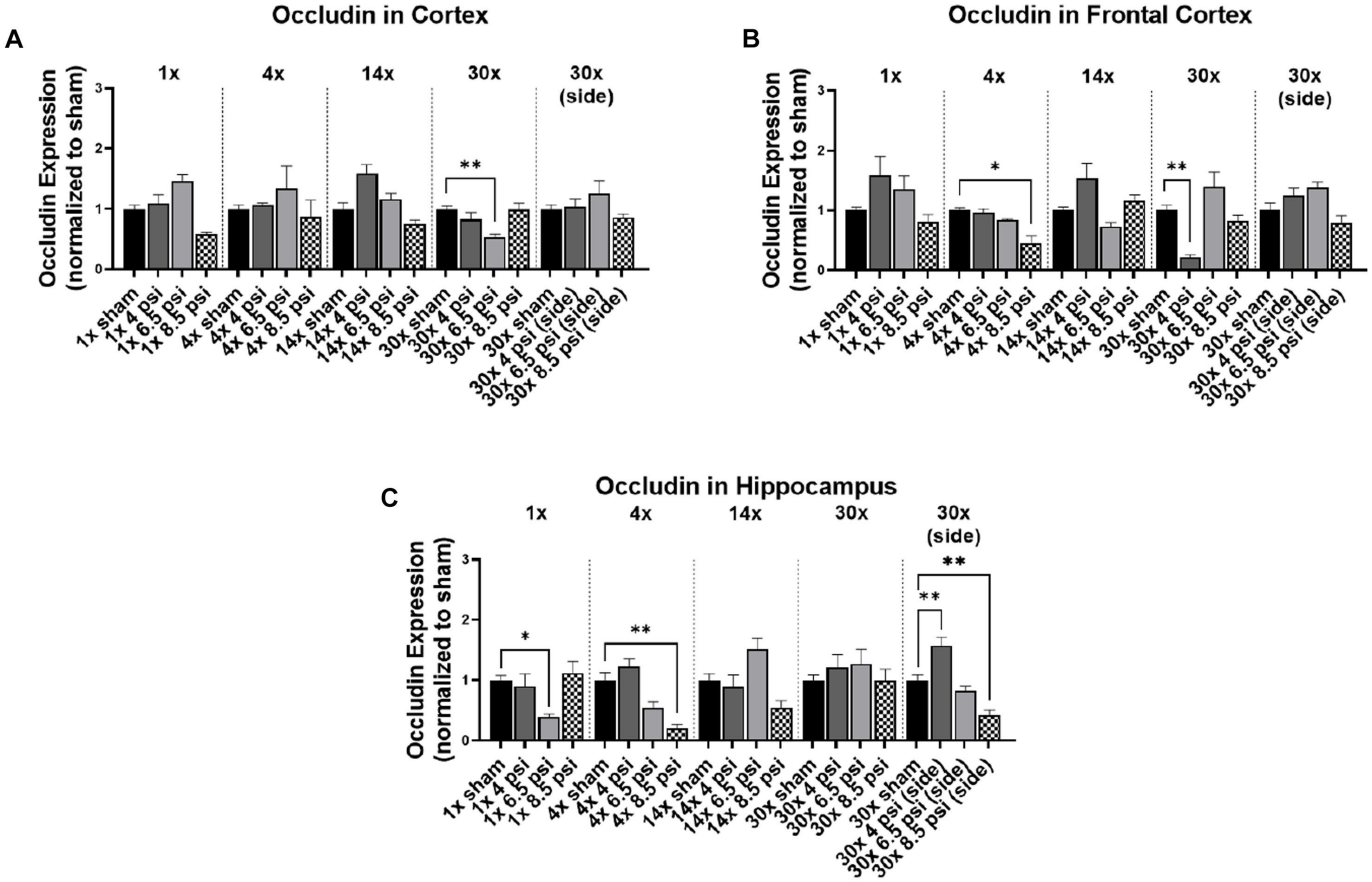
Occludin levels following BOP. Quantification of occludin as measured by Western blot in **(A)** cortex, **(B)** frontal cortex, and **(C)** hippocampus following single and repeated blast. Values normalized to respective shams with same number of exposures. Data is expressed as mean ± SEM. *p < 0.05, **p < 0.01.

The tight junction protein occludin is an important component of the blood–brain barrier. In the cortex, occludin was significantly reduced (~47%) following 30x-6.5 psi exposures from the front (Figure 1A). In the frontal cortex it was also significantly reduced following 4x-8.5 psi exposures (~54%) and 30x-4 psi from the front (~77%) (Figure 1B). In the hippocampus, occludin was significantly reduced following 1x-6.5 psi (~61%), 4x-8.5 psi (~80%), and 30x-8.5 psi exposures from the side (~57%), but significantly increased following 30x-4 psi exposures from the side (~57%) (Figure 1C).

### 3.2. Claudin-5

**Figure 2:**
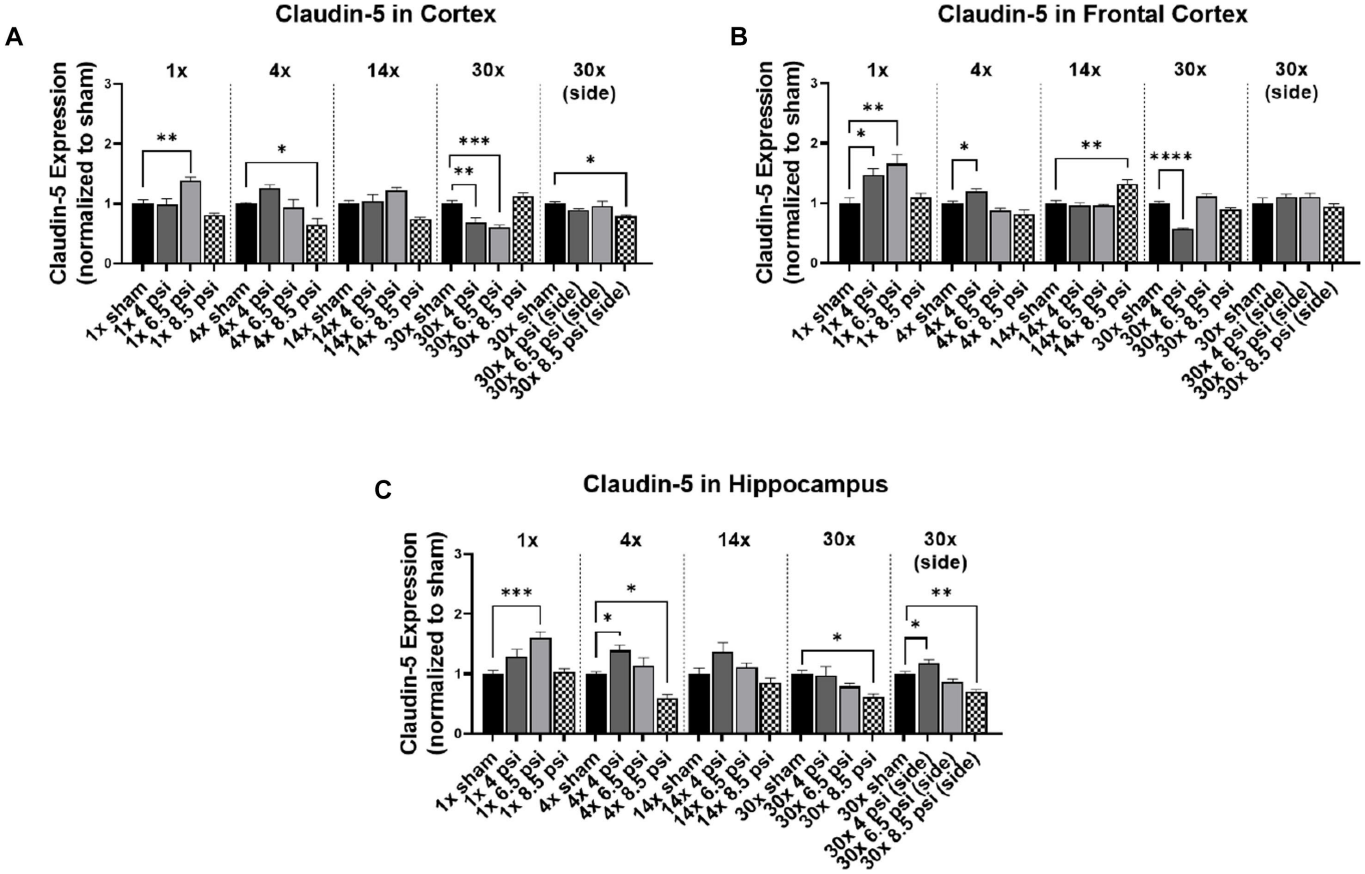
Claudin-5 level following BOP. Quantification of claudin-5 as measured by Western blot in **(A)** cortex, **(B)** frontal cortex, and **(C)** hippocampus following single and repeated blast. Values normalized to respective shams with same number of exposures. Data is expressed as mean ± SEM. *p < 0.05, **p < 0.01, ***p < 0.001, ****p < 0.0001.

**Figure 3:**
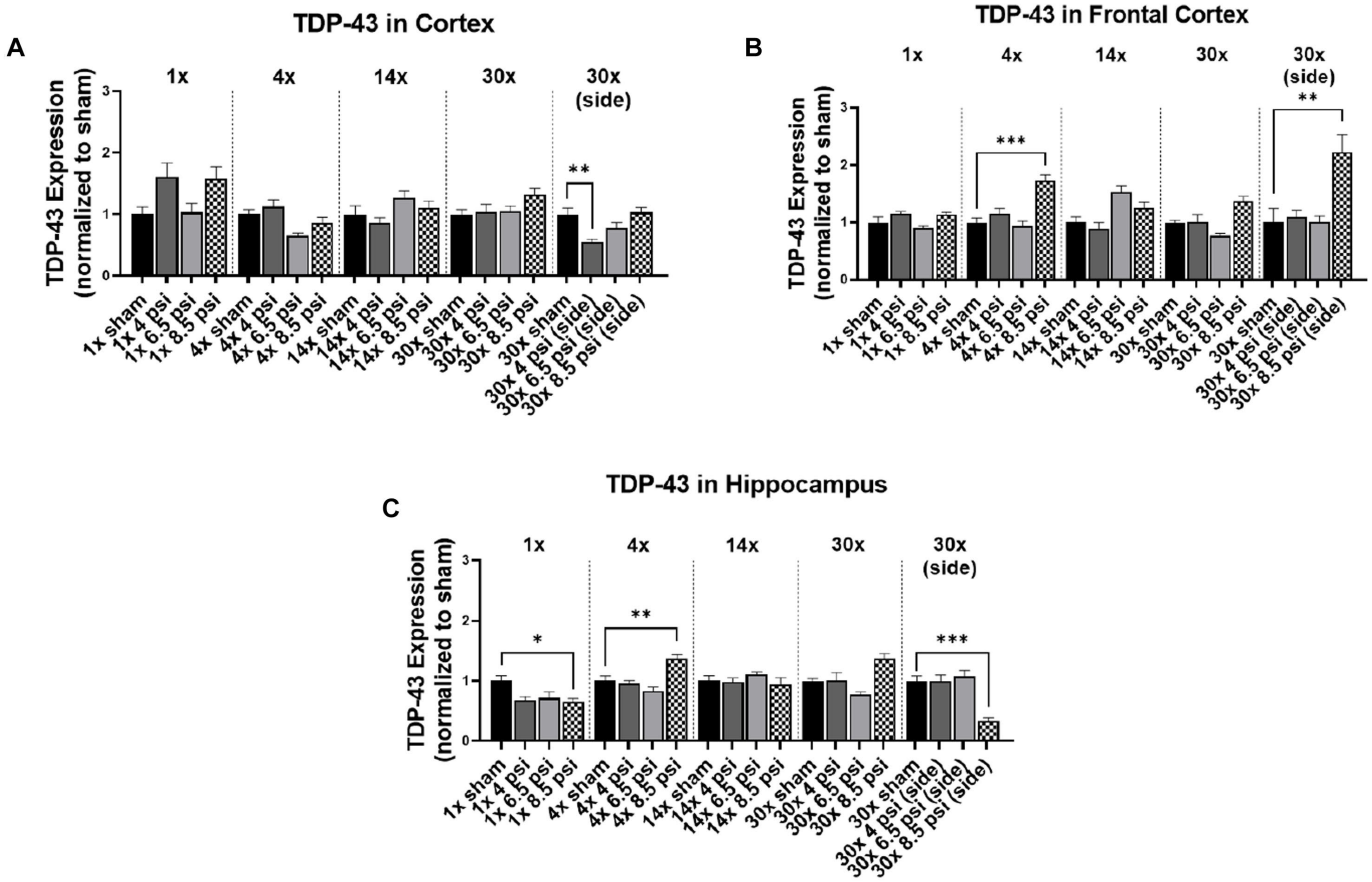
TDP-43 level following BOP. Quantification of TDP-43 as measured by Western blot in **(A)** cortex, **(B)** frontal cortex, and **(C)** hippocampus following single and repeated blast. Values normalized to respective shams with same number of exposures. Data is expressed as mean ± SEM. *p < 0.05, **p < 0.01, ***p < 0.001.

Claudin-5 is another tight junction protein that was assayed in different brain regions. In the cortex, claudin-5 was significantly reduced following 4x-8.5 psi (~36%), 30x-4 psi (~31%) and 6.5 psi (~40%) exposures from the front, and 30x-8.5 psi exposures from the side (~21%) (Figure 2A). However, claudin-5 was significantly increased following a 1x-6.5 psi exposure (~39%) (Figure 2A). In the frontal cortex, claudin-5 is significantly increased following 1x-4 psi (~47%) 6.5 psi (~66%), 4x-4 psi (~20%), and 14x-8.5 psi (~32%) front exposures (Figure 2B). However, it was significantly reduced following 30x-4 psi exposures from the front (~43%). In the hippocampus, claudin-5 was significantly increased following 1x-6.5 psi (~60%), 4x-4 psi (~39%), and 30x-4 psi exposures from the side (~18%). It was significantly reduced following 4x-8.5 psi (~40%) and 30x-8.5 psi exposures from the front (~61%) and side (~29%) (Figure 3C).

### 3.3. TDP-43

The effect of blast on TDP-43 expression, which is normally very tightly regulated in the brain, are shown in Figure 3. In the cortex, TDP-43 was significantly reduced following 30x-4 psi exposures from the side (~45%) (Figure 3A). In the frontal cortex, TDP-43 was significantly increased following 4x-8.5 psi (~73%) and 30x-8.5 psi exposures from the side (~123%) (Figure 3B). In the hippocampus, TDP-43 was significantly decreased following 1x-8.5 psi (~35%) and 30x-8.5 psi exposure from the side (~66%), but significantly increased following 4x-8.5 psi exposures (~37%) (Figure 3C).

### 3.4. Piezo2

**Figure 4:**
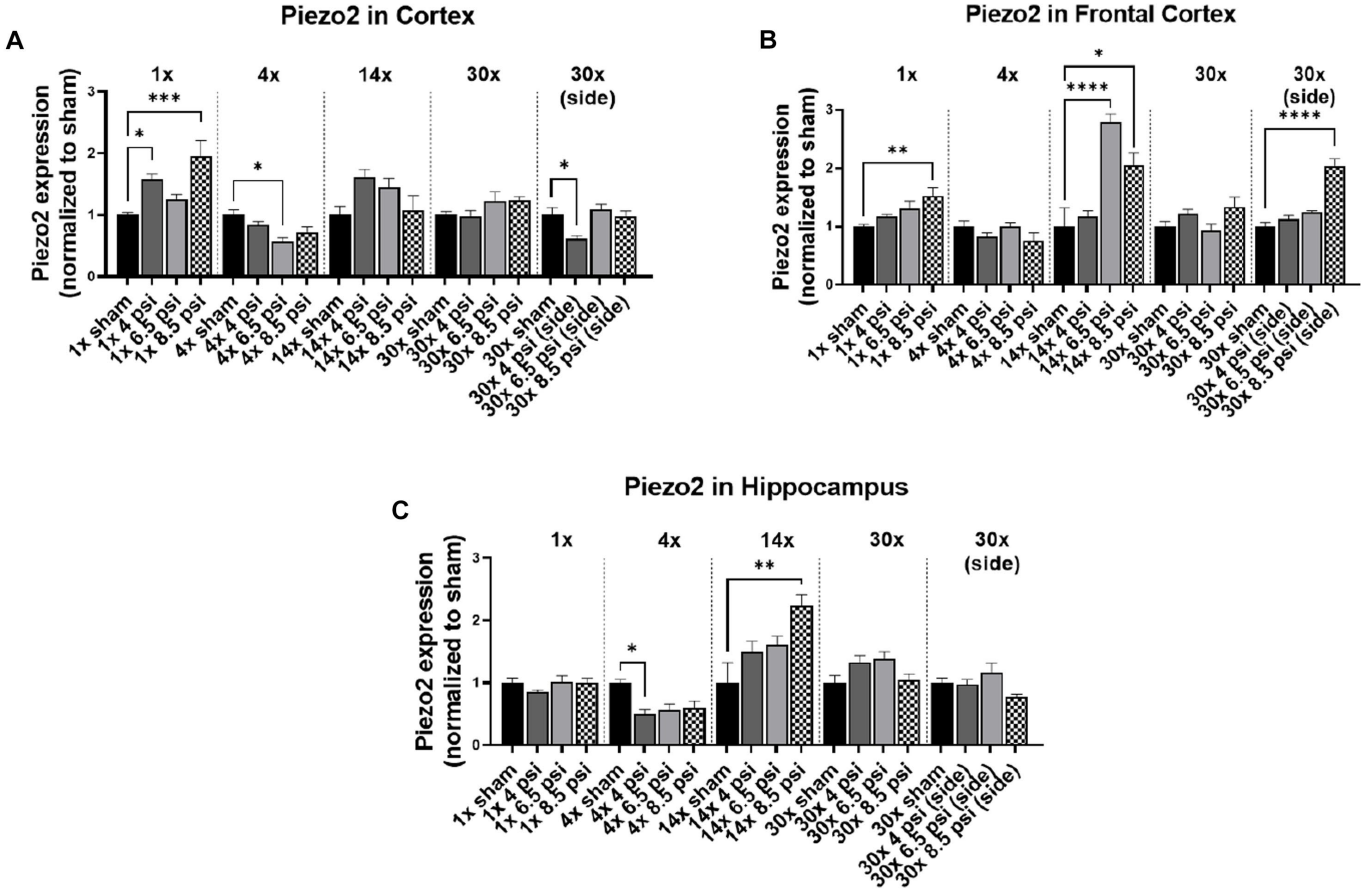
Piezo2 level following BOP. Quantification of piezo2 as measured by Western blot in **(A)** cortex, **(B)** frontal cortex, and **(C)** hippocampus following single and repeated blast. Values normalized to respective shams with same number of exposures. Data is expressed as mean ± SEM. *p < 0.05, **p < 0.01, ***p < 0.001, ****p < 0.0001.

The mechanosensitive protein, piezo2, was assayed using Western blot, to determine whether blast exposure causes alterations mechanosensitive receptors (Figure 4). In the cortex, piezo2 was increased following 1x-4 psi (~58%) and 8.5 psi (~95%) exposures but decreased following 4x-6.5 psi (~44%) and 30x-4 psi exposures from the side (~39%) (Figure 4A). In the frontal cortex, piezo2 was significantly increased following 1x-8.5 psi (~52%), 14x-6.5 psi (~179%), 8.5 psi (~105%), and 30x-8.5 psi exposures from the side (~104%) (Figure 4B). In the hippocampus, piezo2 was significantly reduced following 4x-4 psi (~50%) exposures but was significantly increased following 14x-8.5 psi exposures (~124%) (Figure 4C).

### 3.5. GFAP

**Figure 5:**
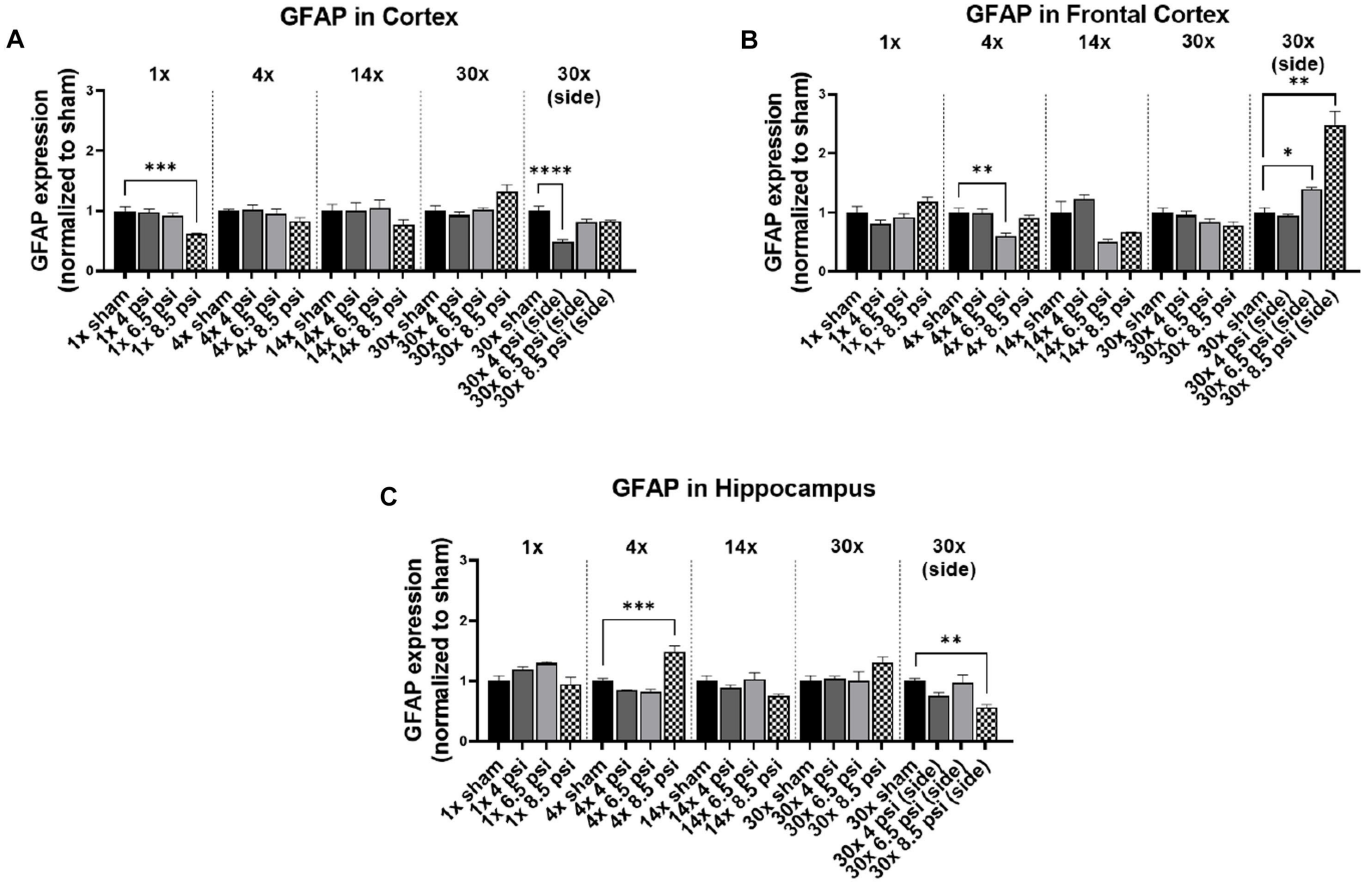
GFAP level following BOP. Quantification of GFAP as measured by Western blot in **(A)** cortex, **(B)** frontal cortex, and **(C)** hippocampus following single and repeated blast. Values normalized to respective shams with same number of exposures. Data is expressed as mean ± SEM. *p < 0.05, **p < 0.01, ***p < 0.001, ****p < 0.0001.

The astrocyte marker, GFAP is associated with inflammation and is abundantly present in astrocytes that maintain blood–brain barrier integrity. In the cortex, GFAP was significantly reduced following 1x-8.5 psi (~38%) and 30x-4 psi (~51%) exposures from the side (Figure 5A). In the frontal cortex, GFAP was significantly reduced following 4x-6.5 psi exposures (~40%) but increased following 30x-6.5 (~39%) and 8.5 psi (~148%) exposures from the side (Figure 5B). In the hippocampus, GFAP was significantly reduced following 30x-8.5 from the side (~43%) but increased following 4x-8.5 psi (~48%) (Figure 5C).

## 4. Discussion

Based on previous pre-clinical and clinical studies (10, 11, 13, 16), we focused on defining the effect of relBOP on the tight junction proteins, claudin-5 and occludin, the levels of which are altered with any injury compromising BBB (13), DNA and RNA binding protein TDP-43, which has altered expression and localization in the brain following TBI (12), and the mechanosensitive protein piezo-2 (17) cortex, frontal cortex, and hippocampus region of the brain following exposures to blast overpressures of 4, 6.5, and 8.5 psi repeated 1x, 4x, 14x, and 30x, with 30x in both frontal and side orientations. In our previous studies, we described the neuropathological effects of blast overpressure based upon intensity, orientation, and frequency of blast overpressure equal to or above 13 psi. However, in training environments Warfighters are also typically repeatedly exposed to intensities of BOP lower than 13 psi, which has prompted growing concern over the potential ill effects on Warfighters’ wellness for operational readiness in the short term and continued operational readiness over the course of a military career (18). The majority of these overpressure exposures elicit sub-concussive pathology inconsistently within different regions of the brain, making it hard to diagnose and identify. Therefore, in this study we assessed the effect of repeated sub-threshold level blast overpressures on different regions of the brain.

Significantly decreased claudin-5 and occludin levels were seen with the 4x-8.5 psi blast group in the cortex and hippocampus, whereas in the frontal cortex the level of claudin-5 significantly decreased only after 30x-8.5 psi repeated blast exposures. Similarly, occludin significantly decreased with 1x-6.5 psi blast exposure in the hippocampus, after 4x-8.5 psi repeated blast in the frontal cortex, and after 30x-6.5 psi in the cortex. The decrease in levels of claudin-5 and occludin can likely be associated with the compromised BBB observed after repeated injury-inducing insults to the brain (13, 19–21). On the contrary, there was a significant increase in the level of claudin-5 after 1x-4 psi and 6.5 psi blast exposures as well as 4x-4 psi blast exposures in all three regions of the brain. This initial increase in the level of claudin-5 and occludin could be part of the repair process to maintain homeostasis of the brain to prevent further damage and potentially promote the restoration of BBB integrity (16). The time-dependent differential expression of these proteins is not unexpected since these proteins are also reported to be transcriptionally regulated at different times post-exposure (13). In the serum assessment of these markers, Agoston et al., have shown differences between student and instructor population for claudin-5 and occludin levels, while instructor population showed an initial decrease followed by recovery to the baselines, student population showed this only for claudin-5 but not occludin (22). Our results showed a similar response in cortical regions with exposure, frequency of exposure and region dependent differences.

No clear pattern of changes was observed for TDP-43 or GFAP protein levels. For example, TDP-43 significantly decreased in the cortex after 30x-4 psi side blast but there were no observable patterns in the levels of these proteins with any other blast exposure conditions in this study. In the frontal cortex, significantly higher levels were seen at 4x-8.5 psi, whereas in the hippocampus TDP-43 first significantly decreased with 1x-8.5 psi blast and then significantly increased at 4x-8.5 psi blast. Surprisingly, no changes were observed for 14x-and 30x-frontal blast for all the intensities in all regions assessed. With the exposure from the side, TDP-43 in the cortex was significantly decreased at 30x-4 psi. However, there was a significant increase only following 30x-8.5 psi exposures in the frontal cortex, and for the hippocampus there was a significant decrease following 30x-8.5 psi side blast exposures. Most studies, including those from our own laboratory, have reported obvious changes in the levels of TDP-43 and GFAP at pressures higher than 8.5 psi (23, 24). We previously showed a significant increase in TDP-43 at 1x exposure of 10 psi vs. 8.5 psi (14). In serum of breachers or students, GFAP was shown to decrease around day 6–7, but the levels have recovered during the two-week breacher course (25). Similarly, Vorn et al. have showed a similar decreasing trend with low-level repeated blast but recovered to normal over the course of the breaching training (26). Similar reports were published by Agoston et al. for GFAP (22). However, Tate et al., reported increased GFAP levels, where recorded pressure was relatively higher than previously reported studies (27). Similarly, in pre-clinical studies different groups have reported different outcomes with GFAP. For example, similar to our results Sosa et al. showed decrease in GFAP after 6 weeks but increased back to normal as sham group after 8 months following 10.8 psi blast (28). Differently in a ferret model of repeated blast exposure Schwerin et al. have shown strong GFAP immunoreactivity at 4 weeks after 4 blast injuries and more subtle reactivity at 1 week with single blast exposure (29). However, it is important to note that in these studies either animals are exposed to 20 psi pressure wave which is much higher than what we used, or the acute phase is considered as 6 weeks which is much longer time than our study. Similarly, another pre-clinical study performed on rat showed increased expression of GFAP in the thalamus at 4 and 7 days and decrease at 11 days with numerous apoptotic cells scattered all over the brain tissue at 30 psi single blast overpressure (30). These pre-clinical and clinical data indicates that these markers are either exposure intensity or time-dependent or recovers to baselines based on compensatory mechanisms, similar to this study. Thus, there is also a possibility that the changes in these proteins are not apparent at lower pressures, even with repeated blast. This, however, requires further investigation with techniques like immunohistochemistry, because the alterations in the expression and regulation of these proteins could also be cell-specific. Notably for TDP-43, cytoplasmic localization is indicative of progression of injury regardless of their expression level estimates using Western blot (21).

Piezo-2 was differently affected among the examined regions of brain. While piezo-2 significantly increased in the cortex after 1x-4 psi and 1x-8.5 psi, it significantly decreased at 4x-6.5 psi, and no changes were observed with 14x-and 30x-frontal blasts at all intensities. There was a significant decrease in piezo-2 in the cortex at 4x-6.5 psi, but the frontal cortex was observed to have a significant increase at 1x-8.5 psi. A significant increase in piezo-2 was seen following 14x-6.5 and 8.5 psi frontal blasts and 30x-8.5 psi side blasts. Interestingly, hippocampal levels of piezo 2 decreased following 1x-4 psi blast, and increased following 14x-8.5 psi blasts, whereas no changes were seen with any other exposure groups. Previous studies have shown that piezo2 significantly increased at single 97 kPa (~14 psi) blast exposure (20) and repeated exposure at 10 psi (11). This potentially indicates that the functionality of piezo-2 channel could be altered with blast overpressures of different magnitude and number of blasts. Sensitivity of piezo-2 to pressures like those resulting from BOP can be potentially leveraged to study injury pathogenesis and can potentially be a target for therapeutic and injury mitigation strategies, which should be further investigated.

Overall, the protein expression in our study suggests that the blast related injury differentially affects different regions of the brain. Based upon these data, it is evident that any one single protein could not by itself be a useful biomarker but rather should be used within a panel of markers for an acute setting.

## Data availability statement

The original contributions presented in the study are included in the article/Supplementary material, further inquiries can be directed to the corresponding author.

## Ethics statement

The animal study was approved by Walter Reed Army Institute of Research. The study was conducted in accordance with the local legislation and institutional requirements.

## Author contributions

LH, VS, and JL designed the study and drafted the manuscript. LH, DW, AB, and RA performed the experiments, analyzed the results, and drafted the manuscript. SD drafted the manuscript and reviewed the data. SA made editorial review. All authors contributed to the article and approved the submitted version.

## Funding

This work was supported/funded by work unit number 602115HP.3720.001.A1317 as a part of the “Exposure Standards for Repeated Blast” program is funded by the Defense Health Program managed by Joint Program Committee 5.

## Author disclaimer

Material has been reviewed by the Walter Reed Army Institute of Research. There is no objection to its presentation and/or publication. The opinions or assertions contained herein are the private views of the author, and are not to be construed as official, or as reflecting true views of the Department of the Army or the Department of Defense. Research was conducted under an IACUC-approved animal use protocol in an AAALAC International – accredited facility with a Public Health Services Animal Welfare Assurance and in compliance with the Animal Welfare Act and other federal statutes and regulations relating to laboratory animals. The views expressed in this manuscript are those of the author and do not necessarily reflect the official policy or position of the Department of the Navy, Department of Defense, nor the U.S. Government. This work was supported/funded by work unit number 603115HP.3520.001.A1411. The study protocol was reviewed and approved by the Walter Reed Army Institute of Research/Naval Medical Research Center Institutional Animal Care and Use Committee in compliance with all applicable Federal regulations governing the protection of animals in research. The experiments reported herein were conducted in compliance with the Animal Welfare Act and per the principles set forth in the “Guide for Care and Use of Laboratory Animals,” Institute of Laboratory Animals Resources, National Research Council, National Academy Press, 2011. Some of the authors are Military Service Members [or employees of the U.S. Government]. This work was prepared as part of their official duties. Title 17, U.S.C., §105 provides that copyright protection under this title is not available for any work of the U.S. Government. Title 17, U.S.C., §101 defines a U.S. Government work as a work prepared by a military Service member or employee of the U.S. Government as part of that person’s official duties.
