# Supplementary figures and images for: Differential effects on TDP-43, piezo-2, tight-junction proteins in various brain regions following repetitive low-intensity blast overpressure
Source: Front Neurol. 2023 Oct 9;14:1237647. doi: 10.3389/fneur.2023.1237647 (PMC10593467; doi:10.3389/fneur.2023.1237647)

**
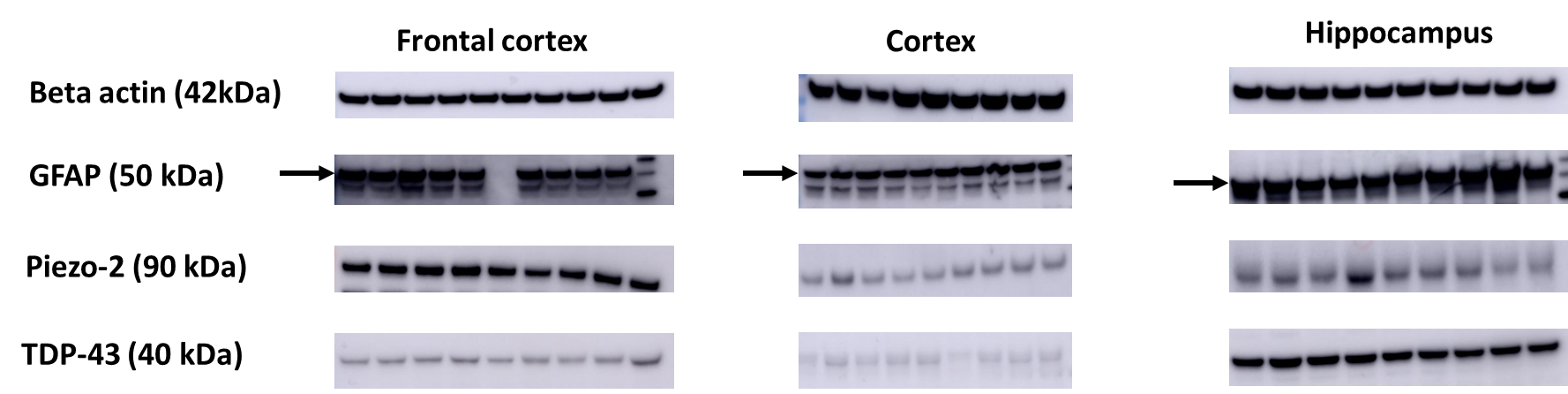
Supplemental material: Representative Blots**

Supplement: Supplementary file 2 [file Table_2.DOCX]
